# Accuracy and precision of visual and auditory stimulus presentation in virtual reality in Python 2 and 3 environments for human behavior research

**DOI:** 10.3758/s13428-021-01663-w

**Published:** 2021-08-03

**Authors:** Ryo Tachibana, Kazumichi Matsumiya

**Affiliations:** 1 Department of Human Information Sciences, Graduate School of Information Sciences, Tohoku University, 6-3-09 Aoba, Aramaki aza, Aoba-ku, Sendai, 9808579 Japan; 2 Department of Applied Information Sciences and Department of Human Information Sciences, Graduate School of Information Sciences, Tohoku University, 6-3-09 Aoba, Aramaki aza, Aoba-ku, Sendai, 9808579 Japan

**Keywords:** Virtual reality, Stimulus presentation, Python 2 & 3, Accuracy & precision, Head-mounted display

## Abstract

Virtual reality (VR) is a new methodology for behavioral studies. In such studies, the millisecond accuracy and precision of stimulus presentation are critical for data replicability. Recently, Python, which is a widely used programming language for scientific research, has contributed to reliable accuracy and precision in experimental control. However, little is known about whether modern VR environments have millisecond accuracy and precision for stimulus presentation, since most standard methods in laboratory studies are not optimized for VR environments. The purpose of this study was to systematically evaluate the accuracy and precision of visual and auditory stimuli generated in modern VR head-mounted displays (HMDs) from HTC and Oculus using Python 2 and 3. We used the newest Python tools for VR and Black Box Toolkit to measure the actual time lag and jitter. The results showed that there was an 18-ms time lag for visual stimulus in both HMDs. For the auditory stimulus, the time lag varied between 40 and 60 ms, depending on the HMD. The jitters of those time lags were 1 ms for visual stimulus and 4 ms for auditory stimulus, which are sufficiently low for general experiments. These time lags were robustly equal, even when auditory and visual stimuli were presented simultaneously. Interestingly, all results were perfectly consistent in both Python 2 and 3 environments. Thus, the present study will help establish a more reliable stimulus control for psychological and neuroscientific research controlled by Python environments.

## Introduction

Virtual reality (VR) has attracted much attention as a new methodology for scientific research. As described in Cipresso et al. (2018), VR technologies immerse us in a virtual environment and enable us to interact with it. These features help establish more natural environments for experiments in three dimensions (3D), where participants can see, hear, and behave as in the real world, enhancing the ecological validity of research (Parsons, 2015). Such environments have also been referred to as “ultimate Skinner box” environments (Rizzo et al., 2004; Wilson & Soranzo, 2015). Since researchers can control stimuli and procedures that are not easily controllable in the real world, VR has been applied in studies on rehabilitation, therapy, and social interaction (Pan & de Hamilton, 2018; Parsons, 2015). In particular, modern VR head-mounted displays (HMDs), such as HTC Vive and Oculus Rift, allow the presentation of complex and dynamic stimuli that can achieve higher ecological validity (close to daily life) and be subject to more experimental control (Loomis et al., 1999; Parsons, 2015). Indeed, a recent study has indicated that the VR HMD of HTC Vive enables the measurement of visual cognition performance, such as visual attention and working memory capacity, as reliably as a cathode-ray tube (CRT) display (Foerster et al., 2019).

## Accuracy and precision of stimulus presentation

As the accuracy and precision of stimulus presentation have been critical for psychological and neuroscience research, millisecond stimulus control should be considered in VR studies. If experiments are performed with low accuracy and precision or untested apparatus, it is difficult to collect replicable data. In particular, in psychological or neuroscientific experiments, stimuli such as visual (e.g., geometric figure, pictures, and animations) and auditory (e.g., tone sound, voice, and music) information should be presented to participants for a set duration and timing with millisecond accuracy and precision. Accuracy in stimulus presentation is measured in reference to the constant error, which is the lag or bias from the true value (designed duration of stimulus) in the experimental procedure, whereas precision is measured in reference to the trial-to-trial variability, that is, jitter or variable error (standard deviation) of stimulus presentation (Bridges et al., 2020). If a picture and a sound with a transistor-transistor logic (TTL) trigger used for event marking in brain activity recording (e.g., EEG, MEG) are presented simultaneously for 100 ms, the stimuli and TTL trigger should ideally be synchronized. Each duration should be 100 ms with no time gap between stimulus onsets (i.e., no time lag). However, the actual stimulus presentation may not be synchronized correctly. There may be a large lag in visual and auditory stimuli from the TTL trigger (low accuracy), and the duration of those stimuli may change unstably to become either shorter or longer than TTL (low precision). The low accuracy and precision do not only collapse the experimental procedure but also disturb the participants’ performance on the task due to unsuitable stimulus onset asynchrony (SOA). This issue can occur in every experiment owing to various hardware- and software-related issues. Thus, even in VR studies, the accuracy and precision in experimental environments should be tested and validated to obtain well-controlled methods with replicability (Plant, 2016).

## Hardware devices for standard laboratory experiments

Traditionally, the risk of low accuracy and precision has been remarkably improved by proper hardware devices in standard laboratories for two-dimensional (2D) environments, but not in VR. For visual stimulus presentation, CRT or low-latency liquid crystal display (LCD) monitors have been used in traditional experiments. CRT displays are still the best choice because of their quick response. Every pixel on the phosphor screen of a CRT is illuminated from top left to bottom right by an electron beam, and its illuminance reaches a maximum level quite rapidly (almost no persistence) (Elze, 2010). It enables the presentation of a visual stimulus without a millisecond time lag (virtually zero). Furthermore, high-performance LCD monitors have also been used instead of CRT monitors because CRTs are no longer produced. In the past, LCDs did not have a rational response time for stimulus presentation. The time to peak illuminance was too slow in the LCD, causing a delay in stimulus onset. While the electrum beam directly illuminates the phosphor screen on a CRT, a backlight behind a layer of liquid crystal is used in LCD. The illuminating light from the backlight needs to pass through a liquid crystal layer placed between polarizing filters. Currently, specific LCDs used for experiments or high-performance LCDs for gaming provide stable and low-latency environments for visual stimuli (Elze, 2010; Ghodrati et al., 2015) (a latency of a few milliseconds). Presentation of auditory stimuli is more complicated and difficult than that of visual stimuli (Reimers & Stewart, 2016). Compared with visual stimuli, the lag of auditory stimuli presentation can be unstable and much longer, although the human temporal resolution for auditory information is more precise than vision (Ghirardelli & Scharine, 2009). To improve the poor auditory stimulus, researchers need to consider various devices for auditory stimuli in experiments: sound cards, audio interfaces, speakers, and headphones. An audio interface or qualified sound card, including analog-to-digital (A/D) or digital-to-analog (D/A) converters, are generally equipped to generate auditory stimuli without sound distortion or noise. In this case, devices always have input or output latency, which causes a much longer time lag than in visual stimuli (Kim et al., 2020). Speakers or headphones, which are also used to present auditory stimuli to participants, also have time lags. Although there may be time lags, recent audio devices that have low or virtually zero latency should be useful for validating the timing lag of auditory stimuli.

## Python software tools for standard laboratory experiments

In addition to the hardware apparatus, specialized software tools are necessary to generate stimuli using millisecond control. Recently, Python has been widely used in scientific research (Muller et al., 2015). Python is an interpreted programming language that has various libraries and high code readability and is easy to make and debug. Over the last decade, many useful Python software tools have been developed to establish specific experiments for psychology and neuroscience (Dalmaijer et al., 2014; Garaizar & Vadillo, 2014; Krause & Lindemann, 2013; Mathôt et al., 2012). Recently, the use of Python tools for experiments has been confirmed via benchmark tests, ensuring that they have robust accuracy and precision in both laboratory and online studies (Wiesing et al., 2020). Bridges et al. (2020) showed that PsychoPy, which is a popular Python package for cognitive experiments, has robust millisecond accuracy and precision even across different operating systems (Windows, macOS, and Ubuntu) and environments (laboratory and online experiments). In laboratory-based studies using PsychoPy, the mean precision of stimulus duration and its lag were less than 1 ms. In online studies, although the results did not achieve the level of lab-based environments, PsychoPy performed the best with under 5-ms precision for auditory and visual stimuli presentation. These studies indicate the useful advantages of Python for achieving millisecond accuracy and precision.

## VR hardware and software

However, although the well-established hardware and software with millisecond reliability as described above is commonly used in psychological and neuroscience research, little is known about the general time/timing accuracy and precision of stimulus presentation in modern VR experiments. Previous studies on VR HMDs including eye tracking have shown that the spatial accuracy of position and orientations are sufficient for general experiments as well as rehabilitation studies (Borrego et al., 2018; Niehorster et al., 2017). Although modern VR HMDs have organic light emitting diode (OLED) displays that provide fast and precise temporal responses for visual stimuli (Cooper et al., 2013; Wiesing et al., 2020), the time accuracy and precision of VR experiments remain unclear. Wiesing et al. (2020) showed that the duration of a visual stimulus controlled by Unreal Engine (a 3D game engine) is stable even with high rendering workload or head movements in VR. Moreover, recent studies using Python API and Unity (a 3D game engine that is used as major software for VR studies) on HTC Vive Pro have suggested that both environments have over 15 ms latency for visual stimuli and over 30 ms latency for auditory stimuli (Le Chénéchal & Chatel-Goldman, 2018). Importantly, Le Chénéchal and Chatel-Goldman (2018) also suggested that Python environments have better timing accuracy (lower time lag) than Unity, and the auditory latency becomes much longer than the visual latency in both Python and Unity.

## The present study

Previous studies were conducted in specific environments (different VR HMDs and software tools such as Unity or Unreal Engine with specific visual and auditory stimuli and procedures), and general time/timing accuracy and frame-by-frame precision in VR experiments have not been proven across modern VR tools in the same procedure. Furthermore, it is still unclear whether psychological and neuroscientific VR experiments controlled by Python have sufficient timing accuracy and precision for stimulus presentation and whether there are differences between Python 2 and 3 versions, although the use of Python in non-VR studies has been increasing, as described above. Clarifying these issues would enable researchers to establish more suitable VR environments that can be validated by millisecond (adjusted within a millisecond) to their own experimental procedures.

The purpose of this study was to empirically evaluate the accuracy and precision of visual, auditory, and audio–visual stimulus presentations with TTL triggers in VR, using modern VR HMDs across Python 2 and 3 environments. Although various software such as Unity or Unreal Engine can be used for VR experiments, most of the programs are not designed for psychological and neuroscientific experiments (Wiesing et al., 2020), except for Vizard. Vizard is a Python-based application from WorldViz for VR development and experimentation (https://www.worldviz.com/vizard-virtual-reality-software). It supports various VR devices and functions for experiments (e.g., stimulus presentation, data collection, synchronization with external devices) in both Python 2 and 3 environments. Python 2 is relatively old, but it is still useful because some third-party packages are only available in Python 2 environments (Rhoads, 2019). In fact, PsychoPy for behavioral studies supports both versions (Peirce et al., 2019). Moreover, we used TTL triggers to strictly evaluate the synchronization between the triggered time/timing from Python and presented stimuli for the VR HMDs. Our method can be used for various experiments with external devices controlled by TTL signals. Especially in experiments with eye tracking or brain recording that require high timing accuracy and precision, the unstable presentation time and timing of stimuli cause incorrect time stamps (unstable onset and offset of stimulus presentations with large jitter) against TTL signals and incorrect activity timelines for real-time recording of biological data (low accuracy and precision). Therefore, evaluation in Python 2 and 3, respectively, is valuable for researchers to reveal the critical differences that can arise in VR stimulus presentation in Python environments (whether there are millisecond differences). The evaluation also contributes to the understanding of the levels of accuracy and precision of the stimulus control provided by Python in VR experiments, compared with the previous evaluation studies of different environments such as Unreal Engine (C++ language) (Wiesing et al., 2020).

## Experiment 1: Visual stimulus presentation

In Experiment 1, the accuracy and precision of visual stimuli in VR developed in Python environments were evaluated using major VR HMDs such as HTC and Oculus. To examine actual stimulus presentation that is synchronized with the refresh rate of VR devices (i.e., v-sync), stimulus duration was controlled frame by frame (i.e., 11.11 ms per frame in 90-Hz HMDs) (cf., Wiesing et al., 2020). In addition, TTL triggers through serial ports were also sent from the same Python program during the visual stimulus presentation (Bridges et al., 2020). Sending the TTL trigger as a time stamp allowed the measurement of the time lag between the actual stimulus presentation time and timing and triggered ones; this is the same methodology used in psychological and neuroscience research with external equipment such as brain activity recording.

## Method

### Apparatus

#### Experiment software settings

The stimulus presentation in VR was controlled using the Vizard 6 (64 bit) software (Vizard 6.3, WorldViz, USA) and the Vizard 7 (64 bit) software (Vizard 7.0, WorldViz, USA) on a laptop PC (Experiment PC) equipped with an Intel Core i7-10750H (2.6 Hz), Windows 10 operating system (64 bit), 16 GB RAM, and an NVIDIA GeForce RTX 2070 video card (Alienware m15 R3, DELL, USA). The reason Vizard software was used is that it is currently the only Python software that supports psychological and neuroscientific VR studies with useful functions for stimulus control. Researchers can perform and compare VR experiments in both Python 2 and 3 environments directly by Vizard 6 and 7. In experiments using the Python 2 environment, Vizard 6 was used to generate and present a visual stimulus, as it is based on Python 2.7.12, whereas in experiments using Python 3, Vizard 7 was used as it is based on Python 3.8.0. These two environments enabled us to examine whether the different major versions of the Python language affect stimulus control in VR. The code was made using the Python 2 to 3 conversion tool (Python 2 to 3 tool, WorldViz, USA: https://docs.worldviz.com/vizard/latest/Python2to3.htm#2To3Tool) to maintain the same coding structure between the two versions. The vertical synchronization (v-sync) setting of display was always turned on in both Vizard 6 and 7 to control the stimulus presentation refresh rates, using the “viz.vsync()” function. The USB power saving settings of the Experiment PC were disabled to maintain high-performance connections between the PC and the VR HMDs.

#### VR head-mounted display settings

We used two different HMDs for stimulus presentation in VR in each experiment: an HTC Vive Pro HMD (HTC Vive Pro, HTC, Taiwan; 2880 × 1600 pixel resolution (1440 × 1600 per eye), 90-Hz refresh rate), and an Oculus Rift HMD (Oculus Rift, Facebook Technologies, USA; 2160 × 1200 pixel resolution (1080 × 1200 per eye), 90-Hz refresh rate). The “Motion Smoothing” system in SteamVR for HTC Vive Pro (SteamVR 1.15.12, Valve, USA) was disabled because the frame smoothing systems in modern VR HMDs can change the frame rate automatically and disturb the stimulus presentation based on the programmed frame rate (90 Hz) during VR experiments. Due to the “Asynchronous Space Warp” system on Oculus Rift, a frame smoothing system in Oculus devices that works automatically even if it is turned off by the Oculus Rift software (Oculus Debug Tool, Facebook Technologies, USA), we measured the luminance change of Oculus HMD by turning “CRT Refresh Correction” on in the Black Box Toolkit.

#### Evaluation device settings

**Fig. 1:**
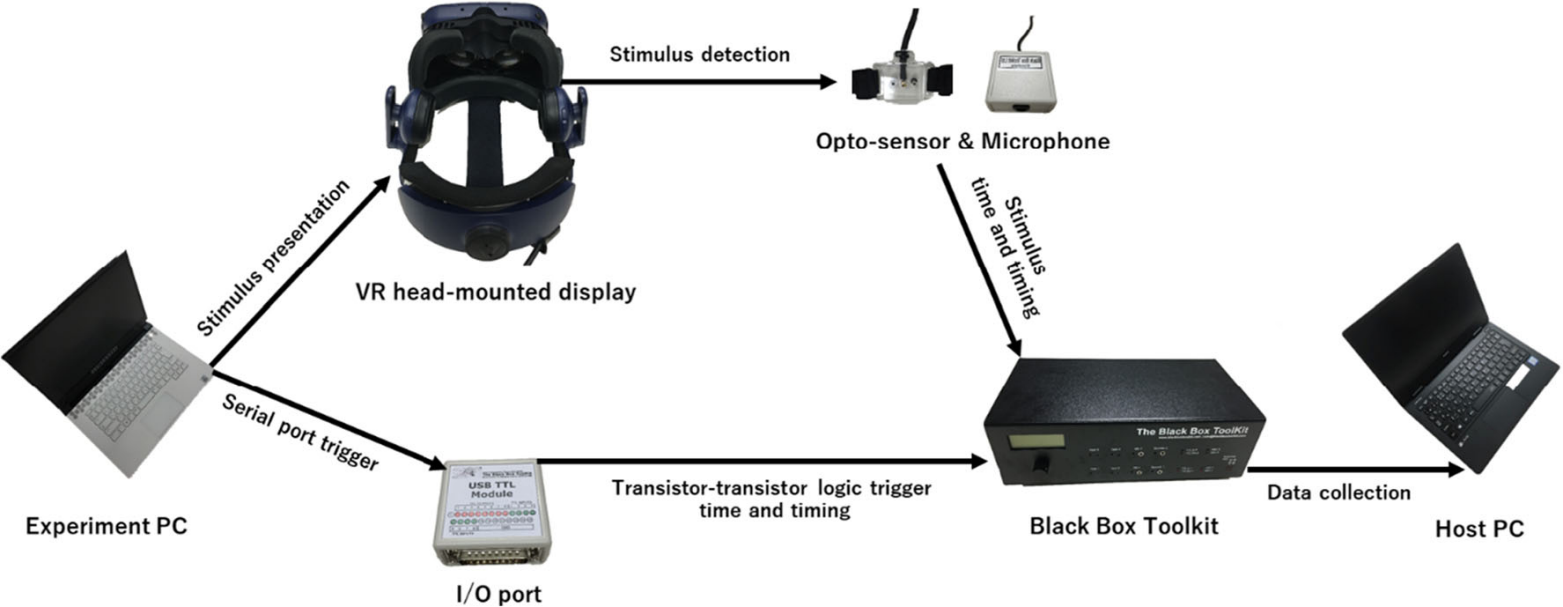
A schematic diagram of evaluation setup

To evaluate the accuracy and precision of visual stimulus presentation in milliseconds, Black Box Toolkit (BBTK) (Black Box Toolkit v2 Elite, The Black Box Toolkit, United Kingdom, 36 channels with 6-kHz sampling rate), which is a special measuring device for stimulus timing accuracy and precision (Bridges et al., 2020; Plant et al., 2004; Wiesing et al., 2020), was used on another independent laptop PC (Host PC) equipped with an Intel Core i7-7Y75 (1.6 Hz), a Windows 10 operating system (64 bit), and 8 GB RAM (Lavie Direct NM, NEC, Japan) (Figure 1). An opto-sensor connected from the Black Box Toolkit was attached to the left lens of the HMDs to measure the luminance changes (BBTK opto-detector sensor, The Black Box Toolkit, United Kingdom). TTL triggers were sent to the BBTK through the I/O port (USB TTL Event Marking Module, The Black Box Toolkit, United Kingdom) connected to the Experiment PC. The PySerial library was used in both Vizard 6 (Python 2) and Vizard 7 (Python 3) environments to establish this serial port connection for TTL triggers (https://pythonhosted.org/pyserial/) (Bridges et al., 2020; Tachibana & Niikuni, 2017). All evaluation tests were conducted and data were collected using Digital Stimulus Capture mode that allowed to measure both the auditory and visual stimuli onsets and offsets with TTL input triggers in BBTK.

In the experiments using the HTC Vive Pro HMD, the “CRT Refresh Correction” tool in BBTK was turned off because the stimulus presentation by HTC’s HMD was measured by frames correctly, as in CRT displays (11.11 ms per frame). Moreover, the “CRT Refresh Correction” tool was turned on during the experiments with Oculus’s HMD due to Asynchronous Space Warp. This setting enabled the measurement and definition of stimulus duration as TTL triggers. While the Asynchronous Space Warp was working, a black blank was inserted automatically after every short refresh of 2–2.5 ms, preventing the measurement of the visual stimulus presentation by frames. The number of short flashes in the HMD depended on the number of frames presented. For instance, Oculus HMD flashed four times for 2–2.5 ms each when the stimulus duration was 4 frames (44.44 ms), and the black blanks were inserted among these short flashes. Hence, the time of visual stimulus in Oculus was measured as the duration from the start of the first frame flash to the end of the last frame flash (cf., Wiesing et al., 2020).

#### Stimuli

Black and white full-background blanks in VR were used as visual stimuli. The black background environment (RGB: 0, 0, 0) was generated using the “viz.clearcolor (0, 0, 0)” function (the arguments “0” correspond to the “0” of each RGB) to change all colors in the VR environment to black on HTC Vive Pro (6.65 cd/m^2^) and Oculus Rift (0.42 cd/m^2^). Similarly, when a black background was generated, the white blank (RGB: 255, 255, 255) in the VR environment was generated using the “viz.clearcolor (1, 1, 1)” (the arguments “1” correspond to the “255” of each RGB) on HTC Vive Pro (116.80 cd/m^2^) and Oculus Rift (78.24 cd/m^2^). The luminance of blanks presented on each HMD was measured by a luminance and color meter (CS-150, Konica Minolta, Japan). In experiments with Python 2 environment, all visual stimuli were generated and controlled by Vizard 6 (Python 2 code). Otherwise, this was done using Vizard 7 (Python 3 code).

#### Procedure

The black-to-white screen transition test, which is a well-established evaluation for stimulus timing accuracy and precision (Garaizar & Vadillo, 2014; Krause & Lindemann, 2013; Tachibana & Niikuni, 2017; Wiesing et al., 2020), was performed in VR. In the experiments, black and white blanks were shown alternately 1000 times in the HMDs. The duration of each blank was 11.11, 22.22, 33.33, 44.44, or 99.99 ms along with 1, 2, 3, 4, and 9 frames of the HMD display, respectively. The durations of these stimuli were controlled by the function “viztask.waitFrame()” for the precise frame number of white and black blanks. The TTL triggers were sent at the onset of each blank. During the tests, visual stimulus presentation and TTL triggers were measured using BBTK. This measurement enabled the analysis of differences between the actual time and timing of stimulus presentation on HMDs and programmed time and timing by TTL in VR. The test was performed in both Python 2 and 3 environments using two HMDs (HTC Vive Pro and Oculus Rift) separately. Thus, 20 tests (two Python environments × two VR HMDs × five stimulus durations) were conducted. The opto-sensor was calibrated using BBTK sensor threshold manager before the experiments.

### Results and discussion

For descriptive statistics analysis, we analyzed the number of presented stimuli (white blank), the average duration of stimulus presentation, and the average time lag and its standard deviation between the onsets of TTL triggers and stimulus presentation (Bridges et al., 2020; Le Chénéchal & Chatel-Goldman, 2018; Reimers & Stewart, 2016) (Table 1). If the number of presented stimuli did not reach the expected count of 1000 (1000 means correct presented number of stimuli synchronized with the frame rates), we excluded it from the analyses of duration and time lag. This data screening enabled us to clarify whether the vertical synchronization worked correctly and what was the sufficient frame number for accurate stimulus presentation in each HMD. Additionally, as shown in Table 1, this screening was performed because the data of stimulus duration and time lag from incorrectly presented number of stimuli (e.g., 53/1000 times in 11.11 ms with Oculus HMD) should not be analyzed with data from correctly presented number of stimuli (1000/1000 times) due to the differences in the sample number.

**Table 1:**
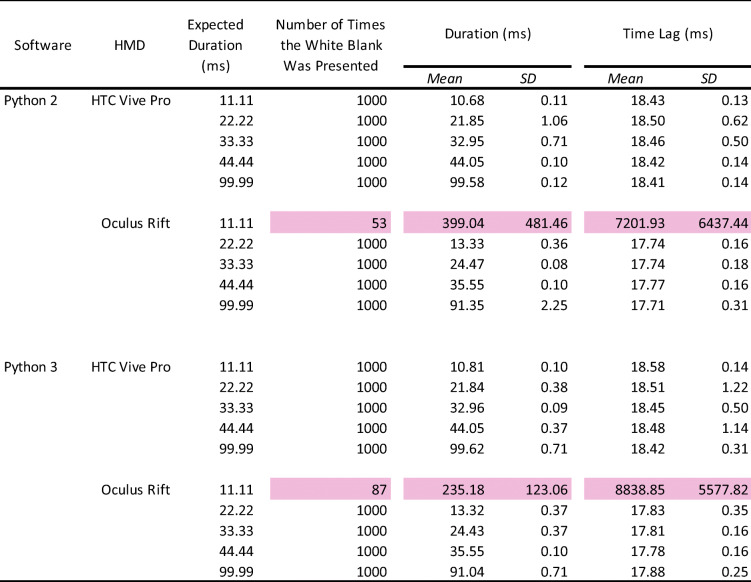
Summary of the number of presented visual stimuli, average duration, and onset lag

The pink-highlighted data represent no accuracy of stimulus presentation. HMD: head-mounted display. SD: standard deviation.

#### Number of stimulus presentations

The white blank was perfectly presented 1000/1000 times with the expected durations, except for the 11.11-ms duration in Python 2 with Oculus Rift (53/1000 times) and Python 3 with Oculus Rift (87/1000 times) environments, indicating that it was difficult to present visual stimuli for one frame accurately.

#### Duration of stimulus presentation

Overall, there were no differences between the Python 2 and Python 3 environments. The average stimulus duration of the Oculus Rift was 8–9 ms shorter than the expected duration. HTC Vive Pro was accurate for all the expected durations. In both HMDs, the standard deviations were less than 1 ms, indicating high precision.

#### Time lag of stimulus presentation

Similar to the duration, there were no differences between Python 2 and Python 3, and the standard deviations were under 1 ms overall. Importantly, there was a 17–18-ms time lag from the TTL trigger to present visual stimulus in every condition, suggesting that it is a constant delay for visual stimuli in VR using Python environments.

## Experiment 2: Auditory stimulus presentation

In Experiment 2, the accuracy and precision of auditory stimuli in VR were evaluated using the same procedure as in Experiment 1.

### Method

#### Apparatus

A microphone (BBTK digital microphone, Black Box Toolkit, United Kingdom) was used instead of an opto-sensor to measure auditory stimulus presentation. The microphone was attached to the left speaker of the HMD. The sound settings of the HMD’s active speaker were activated by SteamVR for HTC Vive Pro and the Oculus software for Oculus Rift. The other apparatus was identical to that used in Experiment 1.

#### Stimuli

We used a pure tone sound (440-Hz sine wave; 44.1-kHz sampling rate; 16-bit depth; 74 dB (A) in HTC HMD, 72 dB (A) in Oculus HMD) as the auditory stimulus. The stimulus was created by the WaveGene software (WaveGene Ver 1.40, Japan: https://efu.jp.net/) and imported into Vizard programs by the “viz.playSound()” function to preload the auditory stimulus.

### Procedure

In the experiment, silence (no sound) and sine-wave sound were presented alternately 1000 times. There was no visual stimulus presentation in the experiments (the background color remained black). The duration of each time was 11.11, 22.22, 33.33, 44.44, or 99.99 ms, as in Experiment 1. These stimulus durations were controlled by the function “viztask.waitFrame()” for the precise frame number of sine waves as well as silences. The TTL triggers were sent at each sound onset. During the experiments, the auditory stimulus presentation and TTL triggers were measured using BBTK. The test was performed in both Python 2 and 3 environments using two HMDs (HTC Vive Pro and Oculus Rift), and 20 tests (two Python environments × two VR HMDs × five stimulus durations) were conducted. The microphone was calibrated using BBTK sensor threshold manager before the experiments.

### Results and discussion

The data were analyzed in the same manner as in Experiment 1 (Table 2).

**Table 2:**
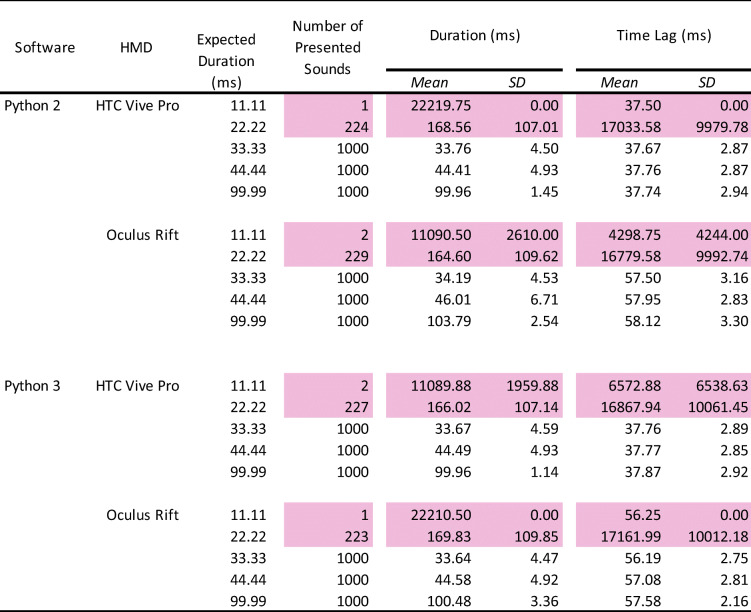
Summary of the number of presented sounds, mean duration, and onset lag

The pink-highlighted data represent no accuracy of stimulus presentation. HMD: head-mounted display. SD: standard deviation.

### Number of stimulus presentations

The pure tone sound was perfectly presented 1000 times, except for the 11.11-ms and 22.22-ms duration. In 11.11 ms, the tone sound was out of control (1 or 2 /1000 times). In these conditions, stimulus presentation by frame rate did not work correctly and the sound was poor with frequent breaks, indicating that the auditory stimulus needed to have a duration of at least 30 ms.

### Duration of stimulus presentation

Overall, there were no differences between Python 2 and Python 3. In addition, the mean duration in both HTC and Oculus HMDs was accurate and almost the same. In contrast to the visual stimulus, the standard deviations were bigger (over 4 ms) in 33.33-ms and 44.44-ms duration. In 99.99 ms, the standard deviations were improved to under 4 ms.

### Time lag of stimulus presentation

Although there were no differences between Python 2 and Python 3, the time lag was larger than in the visual stimulus presentation. In the HTC HMD, there was a constant 38-ms delay from the TTL trigger to present the auditory stimulus. In the Oculus HMD, there was a 57-ms delay. The standard deviations were approximately 3 ms for both HTC and Oculus HMDs, suggesting that the timing accuracy depends on the hardware for the auditory stimulus. These results indicate that the presentation of the auditory stimulus had a lower timing accuracy and precision than the visual stimulus.

## Experiment 3: Audio–visual stimulus presentation

In Experiment 3, the accuracy and precision of audio–visual stimulus presentation in VR were evaluated. This experiment facilitated the measurement of the SOA between auditory and visual stimuli that occurs constantly in VR controlled by Python environments.

### Method

#### Apparatus

The apparatus was identical to that of Experiments 1 and 2. Both the microphone and the opto-sensor were used to measure the audio–visual stimulus presentation simultaneously.

#### Stimuli

The same stimuli as in Experiments 1 and 2 were used.

### Procedure

The procedures of Experiments 1 and 2 were combined. Tests of black-to-white screens with sound were performed in VR. In the test, black and white blanks were shown alternately 1000 times in the HMDs. At the same time as the white blank, a sine-wave sound was presented for the same duration, while there was no sound during the black background presentation. The durations of each blank and sound were 11.11, 22.22, 33.33, 44.44, or 99.99 ms respectively. As in Experiments 1 and 2, the duration of these stimuli was controlled by the function “viztask.waitFrame()” for the precise frame number of both auditory and visual stimuli. TTL triggers were sent at the onset of each blank. During the tests, the visual and auditory stimulus presentations and TTL triggers were measured using BBTK. Similar to Experiments 1 and 2, 20 tests (two Python environments × two VR HMDs × five stimulus durations) were conducted. The microphone and opto-sensors were calibrated by BBTK sensor threshold manager before the experiments.

### Results and discussion

In addition to the analyses used in Experiments 1 and 2 (Appendix Table 9), we analyzed the time lag between visual and auditory stimuli (Table 3).

**Table 3:**
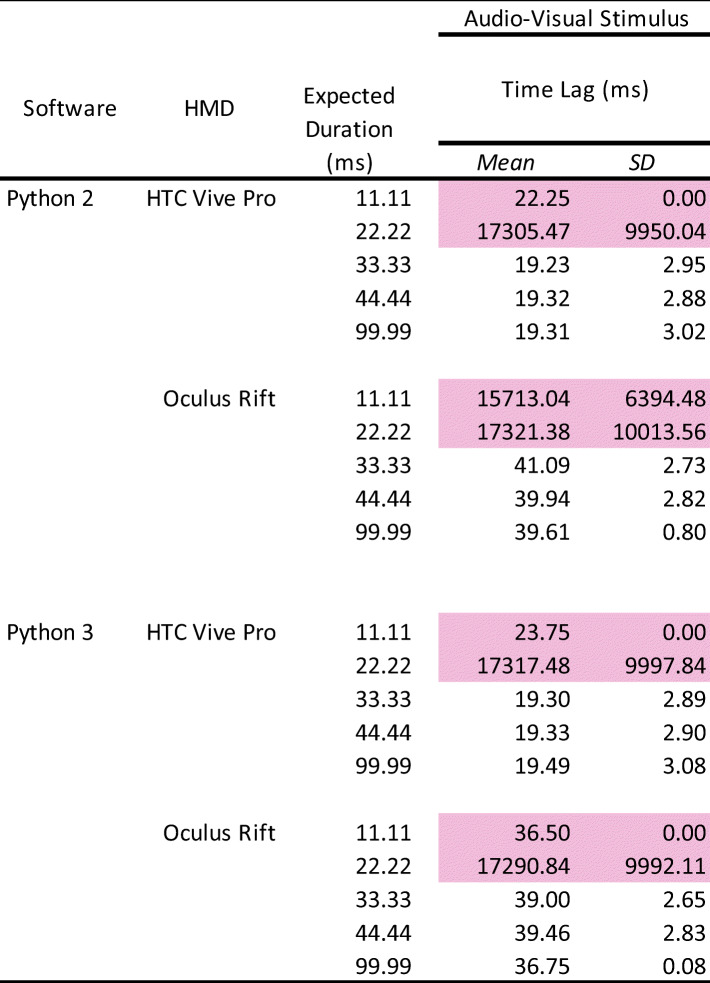
Summary of the time lag between auditory and visual stimuli

The pink-highlighted data represent no accuracy of stimulus presentation. HMD: head-mounted display. SD: standard deviation.

### Number of stimulus presentations

Each presented number of auditory and visual stimuli was the same as in Experiments 1 and 2. The visual stimulus was presented 1000 times, except for the 11.11-ms duration in Python 2 with Oculus Rift (40/1000 times) and Python 3 with Oculus Rift (112/1000 times) environments. For the auditory stimulus, stimulus presentation in the 11.11-ms and 22.22-ms durations did not work correctly, exhibiting disturbed sound in both HTC and Oculus HMDs (1/1000 times).

### Duration of Stimulus Presentation

Overall, the duration of auditory and visual stimuli was the same as in Experiments 1 and 2. There were no differences between Python 2 and 3 in each condition. The mean visual stimulus duration of Oculus Rift was 8–9 ms (1 frame approximately) shorter than the expected duration, whereas HTC Vive Pro had an accurate duration for all expected durations. In both HTC Vive Pro and Oculus Rift, the standard deviations were less than 1 ms, indicating high precision. The mean duration of the auditory stimulus in HTC and Oculus HMDs was accurate and almost the same. Similarly as in Experiment 2, the standard deviations were slightly bigger (over 4 ms) in the 33.33-ms and 44.44-ms durations than in the 99.99-ms duration.

### Time lag of stimulus presentation

Consistent with the results of Experiments 1 and 2, there were stable time lags for both stimuli. For the visual stimulus, a 17–18-ms time lag (SD < 1 ms) occurred in each condition. In the auditory stimulus, the time lag was larger than the visual stimulus presentation: 37-ms time lag in the HTC Vive Pro, and 58-ms time lag in the Oculus Rift. The standard deviations were approximately 3 ms for both HMDs. There were no differences between the Python 2 and 3 environments. These results indicate that there was no negative interaction that could cause a more unstable time lag even if the auditory and visual stimuli were presented simultaneously.

### Time lag between auditory and visual stimuli

There were consistent time lags, depending on the HMDs. The HTC Vive Pro and Oculus Rift had time lags of approximately 19 and 39 ms, respectively, between the auditory and visual stimuli in every condition. The standard deviations of these lags were almost identical, at 3 ms. Similar to the results above, there were no differences between the Python 2 and 3 environments overall.

## Experiment 4A: Visual stimulus presentation with gray-to-gray screen transitions

In Experiments 4 A and B, the accuracy and precision of complex visual stimuli in VR developed in Python environments were evaluated with the method used in Experiment 1. Previous studies have shown that the gray-to-gray transitions of LCD have a different temporal resolution due to their slower rise and fall time of luminance change than black-to-white transitions (Poth et al., 2018). Thus, in Experiment 4A, gray-to-gray screen transition tests in which the screen changes from a gray level to a different gray level were conducted to measure the effects of gray-to-gray changes on visual stimulus presentation in VR HMDs.

## Method

### Apparatus

The apparatus was identical to the apparatus of Experiment 1.

### Stimuli

Full-background blanks in VR with 10% and 90% luminance were used as visual stimuli. The 10% luminance blank was a dark gray level on HTC Vive Pro (11.68 cd/m^2^, generated by viz.clearcolor (0.32, 0.32, 0.32)) and Oculus Rift (7.82 cd/m^2^, generated by viz.clearcolor (0.34, 0.34, 0.34)). The 90% luminance blank was a light gray level on HTC Vive Pro (105.12 cd/m^2^, generated by viz.clearcolor (0.97, 0.97, 0.97)) and Oculus Rift (70.42 cd/m^2^, generated by viz.clearcolor (0.93, 0.93, 0.93)). Similarly to Experiment 1, all visual stimuli were generated and controlled by Vizard 6 (Python 2 code) in experiments with the Python 2 environment. Otherwise, this was done using Vizard 7 (Python 3 code).

### Procedure

The gray-to-gray screen transition test was performed in VR. In the experiments, 10% gray-level and 90% gray-level blanks were shown alternately 1000 times in the HMDs. The duration of each blank was 11.11, 22.22, 33.33, 44.44, or 99.99 ms. The rest of the procedure was the same as in Experiment 1. Twenty tests (two Python environments × two VR HMDs × five stimulus durations) were conducted. The opto-sensor was calibrated using BBTK sensor threshold manager before the experiments.

### Results and discussion

We analyzed the number of presented stimuli (90% gray-level blank), the average duration of stimulus presentation, and the average time lag and its standard deviation between the onsets of TTL triggers and stimulus presentation (Table 4). If the number of the presented stimuli did not reach the expected count of 1000, we excluded it from the analyses of duration and time lag.

**Table 4:**
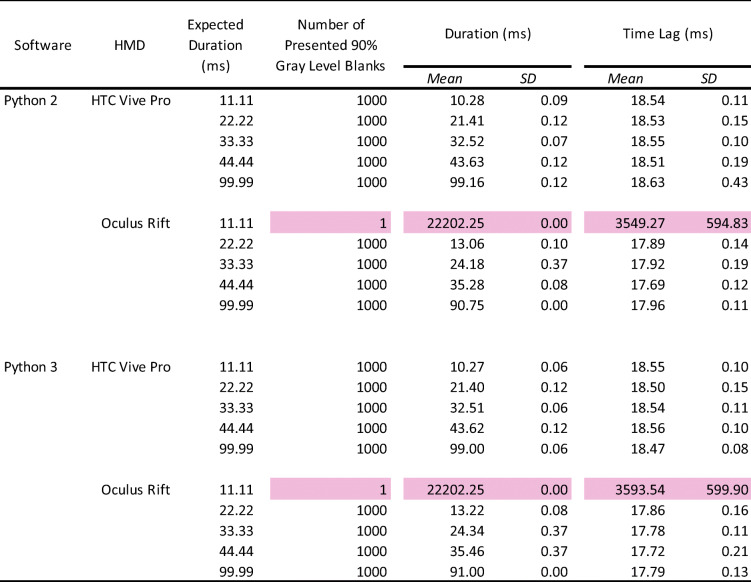
Summary of the number of presented 90% gray-level blanks, average duration, and onset lag

The pink-highlighted data represent no accuracy of stimulus presentation. HMD: head-mounted display. SD: standard deviation.

### Number of stimulus presentations

The 90% gray-level blank was perfectly presented 1000/1000 times with the expected durations, except for the 11.11-ms duration in Python 2 with Oculus Rift (1/1000 times) and Python 3 with Oculus Rift (1/1000 times) environments, indicating that it was difficult to present visual stimuli for one frame accurately. These results were consistent with those of Experiment 1.

### Duration of stimulus presentation

The results were consistent with the results of Experiment 1 (black and white screen transition). There were no differences between the Python 2 and Python 3 environments. The average stimulus duration of the Oculus Rift was 8–9 ms shorter than the expected duration. HTC Vive Pro was accurate for all the expected durations. In both HMDs, the standard deviations were less than 1 ms, indicating high precision.

### Time lag of stimulus presentation

Similar to the duration, there were no differences between Python 2 and Python 3, and the standard deviations were under 1 ms overall. Consistent with the results of Experiment 1, there was a 17–18-ms time lag from the TTL trigger to present visual stimulus in every condition, suggesting that the same time lag occurs in gray-to-gray transitions.

## Experiment 4B: Visual stimulus presentation using complex virtual scene

In Experiment 4B, the accuracy and precision of complex virtual scene as visual stimulus in VR were evaluated. As described in Wiesing et al. (2020), complex visual stimuli such as 3D virtual scenes are quite typical for VR experiments and have a high rendering workload (various 3D objects and textures in a scene) to present as visual stimuli. In contrast, simple stimuli (i.e., black, white, and gray-level blanks) have a minimum rendering workload in VR environments. Evaluation of the stimulus presentation with complex virtual scenes provides evidence on whether there is a longer time lag than with stimuli with low rendering workload.

### Method

#### Apparatus

The apparatus was identical to that of Experiment 4A.

#### Stimuli

**Fig. 2:**
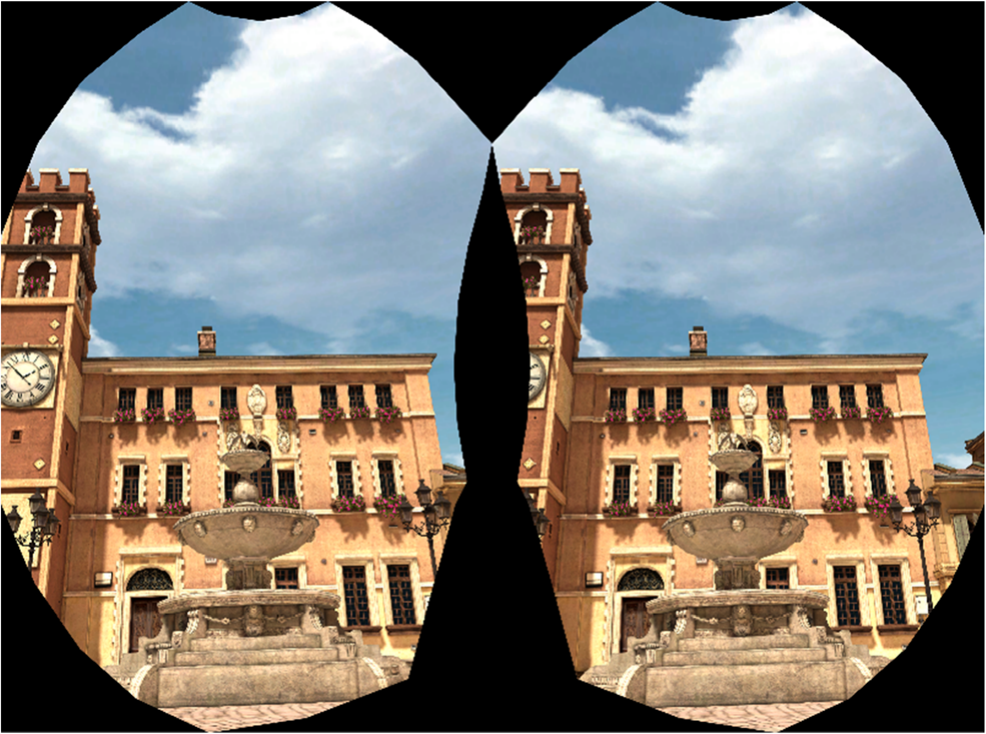
A screenshot of the VR scene in HMDs. This screenshot was taken from Vizard software in HTC Vive Pro with SteamVR

To use a highly realistic VR environment with high rendering workload (Wiesing et al., 2020), a VR scene “piazza” which is implemented in both Vizard 6 and 7 as a standard model was used as a visual stimulus on HTC Vive Pro (31.67 cd/m^2^) and Oculus Rift (23.74 cd/m^2^) (“piazza.osgb”: https://docs.worldviz.com/vizard/latest/#Old_Book/Adding_3D_Models.htm) (Figure 2). The black full-background blank used in Experiment 1 was also used as visual stimulus. All visual stimuli were generated and controlled by Vizard 6 (Python 2 code) in experiments with the Python 2 environment. Otherwise, this was done using Vizard 7 (Python 3 code).

#### Procedure

The procedure was the same as in Experiment 1 except for the visual stimulus. The VR scene was used instead of the white blank. In the tests, the VR scene and black blank were presented alternately 1000 times in the HMDs. The opto-sensor was calibrated using BBTK sensor threshold manager before the experiments.

#### Results and discussion

We analyzed the number of presented stimuli (VR scene), the average duration of stimulus presentation, and the average time lag and its standard deviation between the onsets of TTL triggers and stimulus presentation (Table 5). If the number of presented stimuli did not reach the expected count of 1000, we excluded it from the analyses of duration and time lag.

**Table 5:**
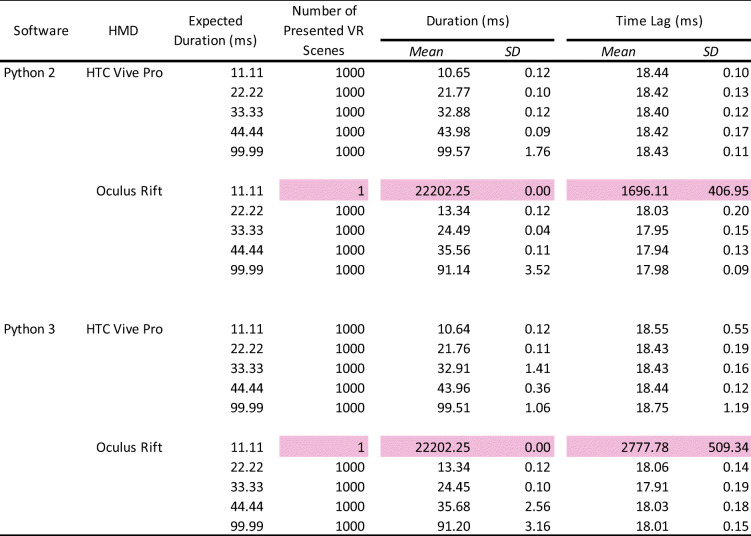
Summary of the number of presented VR scenes, average duration, and onset lag

#### Number of stimulus presentations

The VR scene was perfectly presented 1000/1000 times with the expected durations, except for the 11.11-ms duration in Python 2 with Oculus Rift (1/1000 times) and Python 3 with Oculus Rift (1/1000 times) environments, indicating that it was difficult to present visual stimuli for one frame accurately. These results were consistent with the results of Experiments 1 and 4A.

#### Duration of stimulus presentation

The results were consistent with Experiments 1 (black and white screen transition) and 4A (gray-to-gray screen transition). There were no differences between the Python 2 and Python 3 environments. The average stimulus duration of the Oculus Rift was 8–9 ms shorter than the expected duration. HTC Vive Pro was accurate for all the expected durations. In both HMDs, the standard deviations in 99.99-ms duration were relatively long: 1–3 ms.

#### Time lag of stimulus presentation

There were no differences in the time lags between Python 2 and Python 3, and the standard deviations were under 1 ms overall. Consistent with the results of Experiment 1, there was a 17–18-ms time lag from the TTL trigger to visual stimulus presentation in every condition, suggesting that the same time lag occurs in VR scene presentation.

The pink-highlighted data represent no accuracy of stimulus presentation. HMD: head-mounted display. SD: standard deviation.

## Experiment 5: Complex auditory stimulus presentation

Complex auditory stimuli such as realistic background music (BGM) or sound effects in VR scene are more typically used in VR experiments than a simple tone sound. As the complex visual stimuli tested in Experiment 4B, we evaluated the accuracy and precision of complex auditory stimuli in VR environments using the procedure of Experiment 2.

### Method

#### Apparatus

The apparatus was identical to that of Experiment 2.

#### Stimuli

We used a daily life sounds of a “piazza” (“07035152.wav”; BBC Sound Effects: https://sound-effects.bbcrewind.co.uk/search?q=piazza; 44.1-kHz sampling rate; 16-bit depth; 84 dB (A) in HTC HMD, 78 dB (A) in Oculus HMD) as the auditory stimulus. These are realistic daily life sounds of Piazza Navona which are congruent with the VR scene stimulus in Experiment 4B. The stimulus was edited by the Audacity software (Audacity Ver 3.0.2, The Audacity Team: https://www.audacityteam.org/) to cut the silent part of the sound file out because there were no sounds for the first few seconds in the original file. The stimulus was imported into Vizard programs by the “viz.playSound()” function to preload the auditory stimulus in the same way as in Experiment 2.

#### Procedure

The procedure was the same as in Experiment 2 except for the auditory stimulus. The complex sound was used instead of the pure tone sound. In tests, the complex sound and silence (no sound) were presented alternately 1000 times in the HMDs. The microphone was calibrated using BBTK sensor threshold manager before the experiments.

#### Results and discussion

The data were analyzed in the same manner as in Experiment 2 (Table 6).

**Table 6:**
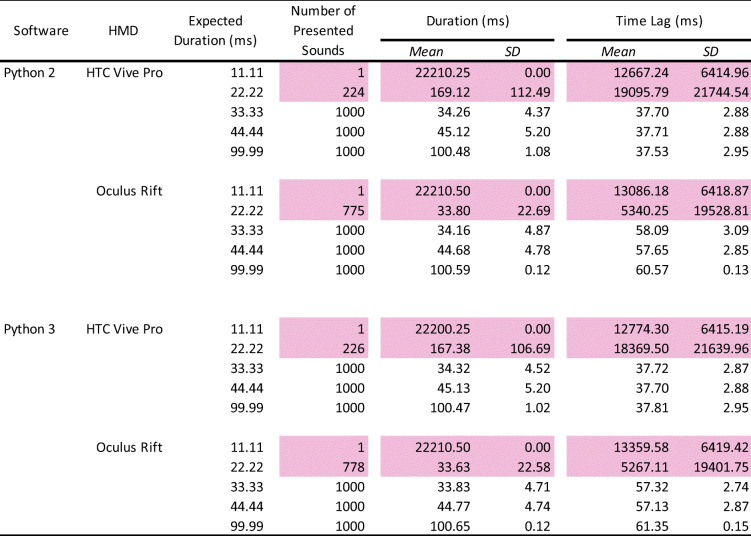
Summary of the number of presented sounds, mean duration, and onset lag

The pink-highlighted data represent no accuracy of stimulus presentation. HMD: head-mounted display. SD: standard deviation.

#### Number of stimulus presentations

The results were consistent with Experiment 2. The complex sound was perfectly presented 1000 times, except for the 11.11-ms and 22.22-ms durations. The complex sound in those durations did not work as expected. In these conditions, the stimulus presentation by frame rate did not work correctly, and the sound was poor with frequent breaks, indicating that the complex sound stimuli needed to have a duration of at least 30 ms for accurate presentation, similar to the pure tone sound.

#### Duration of stimulus presentation

Overall, there were no differences between Python 2 and Python 3 in the duration of stimulus presentation. In addition, the mean durations in both HTC and Oculus HMDs were accurate and almost the same. Consistent with the results of Experiment 2, the standard deviations were bigger (over 4 ms) in 33.33-ms and 44.44-ms durations. In 99.99 ms, the standard deviations were improved to under 4 ms, indicating that the precision of sound stimuli in VR HMDs becomes stable if its duration is over 100 ms.

#### Time lag of stimulus presentation

There were no differences in time lag between Python 2 and Python 3. The time lag was larger than in the visual stimulus presentation in Experiments 1, 4A, and 4B. In the HTC HMD, there was a constant 38-ms delay from the TTL trigger to the auditory stimulus presentation. In the Oculus HMD, there was a 57-ms delay in 33.33-ms and 44.44-ms durations. In the 99.99-ms duration in Oculus HMD, the time lag was a slightly longer 60 ms with under 1-ms jitter. The other standard deviations (in 99.99 ms in HTC HMD, in 33.33 ms and 44.44 ms in both HMDs) were approximately 3 ms, suggesting that the timing accuracy depends on the hardware for the auditory stimulus. Consistent with the results of Experiment 2, these results indicate that the presentation of the auditory stimulus had a lower timing accuracy and precision than the presentation of a visual stimulus.

## Experiment 6A: Audio–visual stimulus presentation with gray-to-gray screen transition

In Experiment 6A, the accuracy and precision of audio–visual stimulus presentation using gray-level screens with complex sound were evaluated. This experiment was conducted to test whether the SOA between auditory and visual stimuli in gray-to-gray screen transitions changed in VR environments.

### Method

#### Apparatus

The apparatus was identical to that of Experiments 4A and 5. Both the microphone and the opto-sensor were used to measure the audio–visual stimulus presentation simultaneously.

#### Stimuli

The same stimuli as in Experiments 4A and 5 were used.

#### Procedure

All procedures were identical to Experiment 3 except for the auditory and visual stimuli. Tests of gray-to-gray screens with complex sound were performed in VR. In 90% gray-level screen presentation, the complex sound was presented simultaneously, whereas there was no sound in 10% gray-level screen. The microphone and opto-sensors were calibrated by BBTK sensor threshold manager before the experiments.

#### Results and discussion

Analyses were performed in the same way as in Experiment 3 (Appendix Table 10) (Table 7).

**Table 7:**
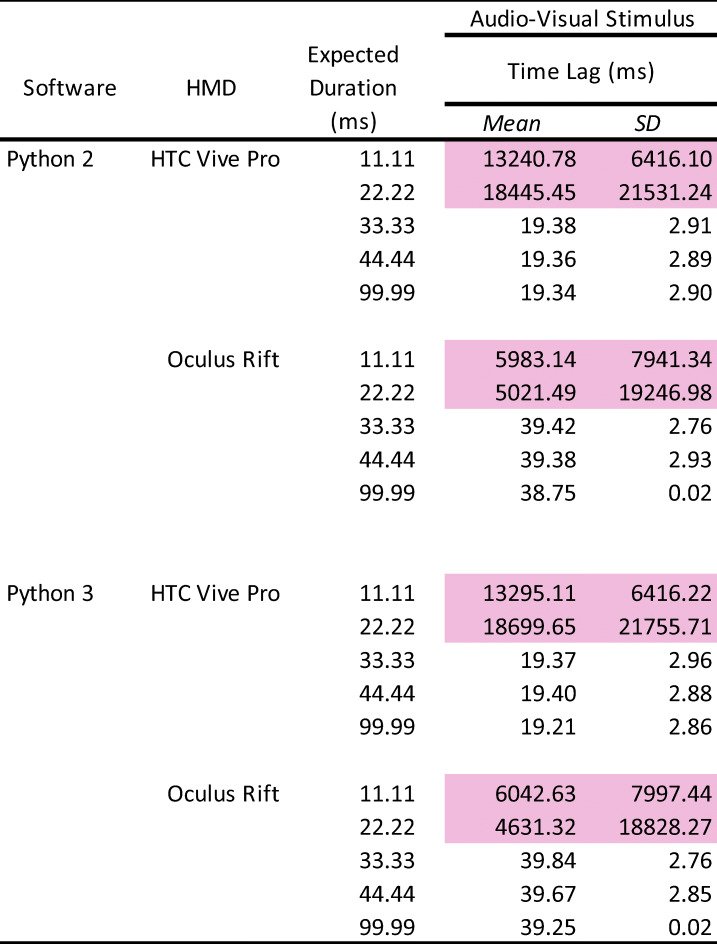
Summary of the time lag between auditory and visual stimuli

The pink-highlighted data represent no accuracy of stimulus presentation. HMD: head-mounted display. SD: standard deviation.

#### Number of stimulus presentations

The numbers of presented number auditory and visual stimuli were consistent with those in Experiments 4A and 5. The visual stimulus was presented 1000 times, except for the 11.11-ms duration in Python 2 with Oculus Rift (445/1000 times) and Python 3 with Oculus Rift (445/1000 times) environments. For the auditory stimulus, stimulus presentation in the 11.11-ms and 22.22-ms durations did not work correctly, exhibiting disturbed sound in both HTC (1/1000 times in 11.11 ms with both Python 2 and 3, 229/1000 times in 22.22 ms with Python 2, 226/1000 times in 22.22 ms with Python 3) and Oculus HMDs (1/1000 times in 11.11 ms with both Python 2 and 3, 785/1000 times in 22.22 ms with Python 2, 795/1000 times in 22.22 ms with Python 3).

#### Duration of stimulus presentation

Overall, the duration of auditory and visual stimuli was the same as in Experiments 4A and 5. There were no differences between Python 2 and 3 in each condition. The mean visual stimulus duration of Oculus Rift was 8–9 ms (approximately 1 frame) shorter than the expected duration, whereas HTC Vive Pro had an accurate duration for all expected durations. In both HTC Vive Pro and Oculus Rift, the standard deviations were less than 1 ms, indicating high precision. The mean duration of the auditory stimulus in HTC and Oculus HMDs was accurate and almost the same. Similarly to Experiments 2 and 5, the standard deviations were slightly bigger (over 4 ms) in the 33.33-ms and 44.44-ms duration than in the 99.99-ms duration (under 4 ms), indicating that the precision of sound stimuli in VR HMDs becomes stable when the duration is over 100 ms, even in the audio–visual stimulus presentation.

#### Time lag of stimulus presentation

Consistent with the results of Experiments 4A and 5, there were stable time lags for both stimuli. For the visual stimulus, a 17–18-ms time lag with 1-ms jitter occurred in each condition. In the auditory stimulus, the time lag was larger than the visual stimulus presentation: 37 ms time lag in the HTC Vive Pro, and 58 ms time lag in the Oculus Rift. The standard deviations were approximately 3 ms for both HMDs. There were no differences between the Python 2 and 3 environments. These results indicate that there was no negative interaction that could cause a more unstable time lag even if the complex sound and gray-level visual stimuli were presented simultaneously.

#### Time lag between auditory and visual stimuli

Similarly to Experiment 3, there were constant time lags, depending on the type of HMD. The HTC Vive Pro and Oculus Rift had time lags of approximately 19 and 39 ms, respectively, between the auditory and visual stimuli in every condition. The standard deviations of these lags were approximately 3 ms except for Oculus HMD in 99.99-ms duration (less than 1 ms), suggesting that Oculus HMD may became more stable if the stimulus duration is over 100 ms. There were no differences between the Python 2 and 3 environments overall.

## Experiment 6B: Audio–visual stimulus presentation using VR scene with complex sound

In Experiment 6B, the accuracy and precision of audio–visual stimulus presentation using a VR scene with complex sounds were evaluated. This experiment facilitated the measurement of more realistic SOA in VR experiments with high rendering workload controlled by Python environments.

### Method

#### Apparatus

The apparatus was identical to that of Experiments 4B and 5. Both the microphone and the opto-sensor were used to measure the audio–visual stimulus presentation simultaneously.

#### Stimuli

The same stimuli as in Experiments 4B and 5 were used.

#### Procedure

The procedure was identical to the procedure of Experiment 6A, except for the visual stimulus. The realistic VR scene used in Experiment 4B was used instead of 90% gray screen stimulus. The black blank was used as visual stimulus instead of 10% gray screen. When the VR scene was presented, the complex sound was presented simultaneously, whereas there was no sound during the black blank presentation. The microphone and opto-sensors were calibrated by BBTK sensor threshold manager before the experiments.

#### Results and discussion

Analyses were performed in the same way as in Experiments 3 and 6A (Appendix Table 11) (Table 8).

**Table 8:**
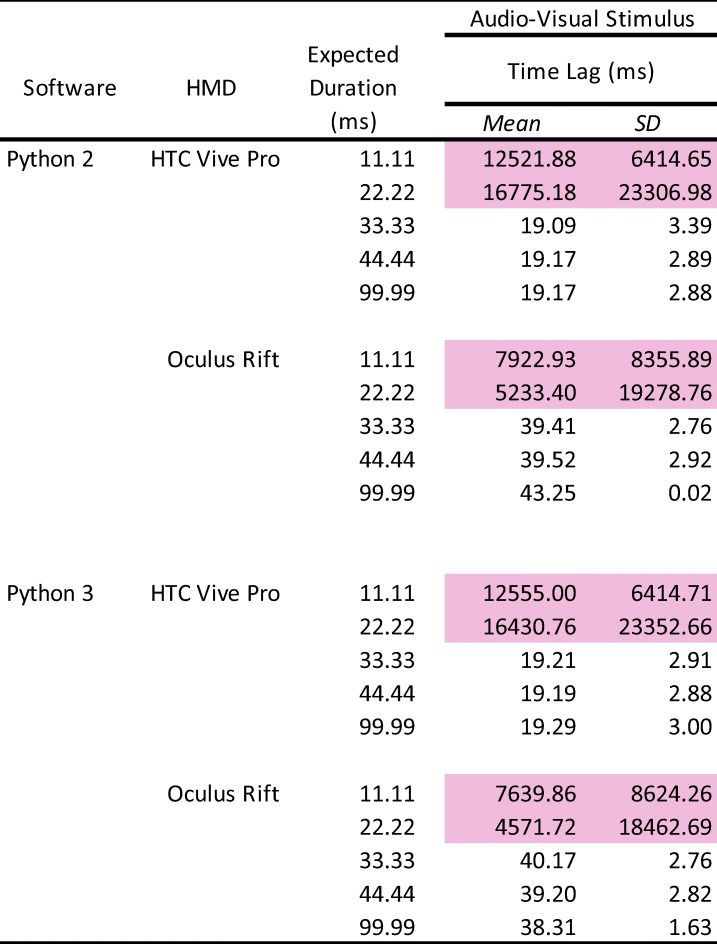
Summary of the time lag between auditory and visual stimuli

The pink-highlighted data represent no accuracy of stimulus presentation. HMD: head-mounted display. SD: standard deviation.

#### Number of stimulus presentations

The presented numbers of auditory and visual stimuli were consistent with those in Experiments 6A. The visual stimulus was presented 1000 times, except for the 11.11-ms duration in Python 2 with Oculus Rift (574/1000 times) and Python 3 with Oculus Rift (526/1000 times) environments. For the auditory stimulus, stimulus presentation in the 11.11-ms and 22.22-ms durations did not work correctly, exhibiting disturbed sound in both HTC (1/1000 times in 11.11 ms with both Python 2 and 3, 284/1000 times in 22.22 ms with Python 2, 292/1000 times in 22.22 ms with Python 3) and Oculus HMDs (1/1000 times in 11.11 ms with both Python 2 and 3, 779/1000 times in 22.22 ms with Python 2, 806/1000 times in 22.22 ms with Python 3).

#### Duration of stimulus presentation

Overall, the durations of the auditory and visual stimuli were the same as in Experiments 4B and 5. There were no differences between Python 2 and 3 in each condition. As in Experiments 1, 3, 4A, 4B, and 6A, the mean visual stimulus duration of Oculus Rift was 8–9 ms (1 frame approximately) shorter than the expected duration, whereas HTC Vive Pro had an accurate duration for all expected durations. In both HTC Vive Pro and Oculus Rift, the standard deviations were less than 1 ms, indicating high precision. The mean duration of the auditory stimulus in HTC and Oculus HMDs was accurate and almost the same as in Experiments 5 and 6A. The standard deviations were also slightly bigger (over 4 ms) in the 33.33 ms and 44.44 ms duration than in the 99.99 ms duration (under 4 ms).

#### Time lag of stimulus presentation

Consistent with the results of Experiments 4B and 5, there were stable time lags for both stimuli. For the visual stimulus, there was a constant 18-ms time lag (less than 1-ms jitter). In the auditory stimulus, the time lag was larger than that of the visual stimulus presentation: 37-ms time lag in the HTC Vive Pro and 58-ms time lag in the Oculus Rift. Similarly to Experiment 5, the standard deviations slightly improved (under 2 ms) in 99.99-ms duration with Oculus HMD, whereas the other standard deviations were approximately 3 ms for both HMDs, suggesting that timing precision for auditory stimulus depends on the hardware devices. There were no differences between the Python 2 and 3 environments. Overall, these results indicate that there was no negative interaction that could cause a more unstable time lag even if the complex VR scene and auditory stimuli were presented simultaneously.

#### Time lag between auditory and visual stimuli

Similarly to Experiment 6A, there were constant time lags, depending on the type of HMD. The HTC Vive Pro and Oculus Rift had time lags of approximately 19 and 39 ms, respectively, between the auditory and visual stimuli in every condition. The standard deviations of these lags were approximately 3 ms except for Oculus HMD in 99.99 ms (around 1 ms), suggesting that Oculus HMD became more stable if the stimulus duration is over 100 ms. There were no differences between the Python 2 and 3 environments overall.

## General discussion

This study systematically evaluated the accuracy and precision of visual, auditory, and audio–visual stimulus presentations in VR controlled by Python environments. The results clearly showed that there is a stable time lag and jitter for each stimulus. The time lag for visual stimulus was approximately 18 ms, whereas for auditory stimuli, it was 37 ms and 58 ms on the HTC Vive Pro and Oculus Rift, respectively. These time lags indicate that there is a considerable 20 ms SOA for HTC’s HMD and 40 ms SOA for Oculus’s HMD when the auditory and visual stimuli are presented simultaneously even when complex stimuli with high rendering workloads were used. For the auditory stimulus, a duration of at least 30 ms (three frames) was required to present it without sound distortion. Importantly, these results were consistent in both Python 2 and 3, indicating no differences between those environments for stimulus presentation.

In Experiment 1, the visual stimulus was presented 18 ms after the TTL trigger in every condition with high precision (onset jitter was under 1 ms approximately). This result is consistent with the previous study that evaluated HTC Vive Pro HMD using the Python API (mean time lag = 18.35 ms, SD = 0.96 ms) (Le Chénéchal & Chatel-Goldman, 2018). In their studies, the time lag was directly tested by native OpenVR wrapped in a Python library to establish a low-level (minimal overload for rendering) Python API, whereas our studies used Vizard, which is a large Python application for VR experiments, on both Python 2 and 3. Thus, the same time lags of 18 ms are caused by the hardware (VR HMD), called the application (or motion)-to-photon latency of the VR HMD (Choi et al., 2018; Le Chénéchal & Chatel-Goldman, 2018). Although the time lag does not show the extremely high accuracy of CRT, the jitter (standard deviation) of stimulus onsets is high and stable (under 1 ms). With the high precision of time lag, researchers can adjust their accuracy by presenting visual stimuli a few frames faster. In VR experiments controlled by Python with a 90-Hz refresh rate, the 18-ms time lag was almost equivalent to two frames (22.22 ms). Adjustment of the two frames achieves improved accuracy for visual stimulus presentation (under 5 ms).

While the accuracy and precision of visual stimuli are consistently stable across every environment, the duration of the visual stimulus depends on the HMD system. In the Oculus HMD, the stimulus duration was one frame shorter than the expected duration. Unfortunately, there was no accuracy for stimulus presentation when the duration was a single frame (11.11 ms). Because of the Asynchronous Space Warp system on Oculus, the frame rate is forced to change and a black blank is inserted automatically in every flash to reduce artifacts during VR running, even if the settings are turned off. This causes the frame rate to decrease from 90 to 45 Hz, and the actual flash of the display (white blank) is reduced. In VR experiments that do not require the millisecond control of stimulus, it may help to run the task on a low-spec graphics card; however, it must be noted that the motion smoothing for VR HMDs interrupts the millisecond control by frames in psychological and neuroscientific experiments. To establish accurate frame control, Oculus Rift Development Kit 2, which is an official SDK for Oculus devices, can be used. For HTC HMDs, motion smoothing is controlled by SteamVR. Because the duration of visual stimulus in HTC Vive Pro is quite accurate and precise frame by frame, it may be more suitable for scientific experiments.

In Experiment 2, the accuracy and precision of the auditory stimulus were lower than those of the visual stimulus. As in the 11.11- and 22.22-ms durations, the sound was distorted and not presented for the expected duration on both HMDs across Python 2 and 3 environments, suggesting that the auditory stimulus is uncontrollable under the 20-ms duration in modern VR HMDs. As the auditory stimulus in VR environments is presented to participants via the headphones integrated in HMDs connected by a DisplayPort or HDMI cable, we cannot use audio interfaces to improve the sound quality, as is usually done in traditional audio hardware setups. Additionally, unlike with libraries for auditory stimuli, which do not report the progress of sound playing, researchers can detect when a graphics card for visual stimulus flipped the frame (Bridges et al., 2020). As for auditory stimulus presentation in 2D environments controlled by Python tools for experiments, there is an 18-ms time lag with 1–3-ms jitter (Krause & Lindemann, 2013; Tachibana & Niikuni, 2017). Although previous studies measured the auditory timing performance directly from the output of the sound card to test the native lag without physical headphones or speakers, the time lag in VR HMDs may still be worse because the latency of recent audio interfaces or headphones is low, and the buffer size of sound can be controlled. Over 33.33 ms, auditory stimulus was presented as per the expected number and duration in all environments. This clearly indicates that 30 ms is the minimum duration required for auditory stimuli without sound distortion in modern VR HMDs. However, there is an approximately 30–60-ms time lag even if the stimulus duration is longer than 30 ms. The time lag varies in HMDs: 37 ms for HTC Vive Pro and 57 ms for Oculus Rift. We should consider these timing delays for each device and adjust them to present the auditory stimulus a few frames faster than the visual stimulus to reduce the time lag to under 4 ms. Moreover, the jitter of duration on 33.33 and 44.44 ms were slightly bigger (over 4 ms) than that on 99.99 ms (under 4 ms). This suggests that the accuracy and precision of auditory stimulus on VR HMDs would be more stable over a duration of 100 ms, although a jitter of less than 5 ms may be sufficient for general experiments. If more millisecond control of the auditory stimulus by Python is required for experiments, the PTB library in PsychoPy 3 can be helpful. This sound library seems to have the lowest time lag and jitter (5-ms time lag with 1-ms jitter) to present the auditory stimulus in Python experimental control (https://www.psychopy.org/api/sound.html). Since it works only in Python 3 environments, Vizard 7 (Python 3) may be required and suitable for presenting more accurate audio stimuli in VR environments.

It is obvious from Experiment 3 that the accuracy and precision of auditory and visual stimuli do not vary even when the stimuli are presented simultaneously on both Python 2 and 3, corresponding to Experiments 1 and 2, where the visual and auditory stimuli were tested independently. The time lag between auditory and visual stimuli also depends on the HMD: 19 ms in the HTC HMD and 39 ms in the Oculus HMD. Interestingly, the jitter was 3 ms. Previous studies on timing accuracy and precision showed that the time lag between auditory and visual stimuli was less than 10 ms with 1-ms jitter in standard laboratory 2D environments with a low-latency LCD monitor, whereas it was over 50 ms with 3–5-ms jitter in web-based environments controlled by PsychoPy on a Windows 10 operating system (Bridges et al., 2020). Compared with laboratory and web-based studies on Python environments, modern VR HMDs may present intermediate accuracy and precision for audio–visual stimulus presentation.

In addition to Experiments 1–3 where the timing accuracy and precision were measured by well-established methods, we expanded the evaluation using complex stimuli in VR environments in Experiments 4–6. As a whole, the results were consistent with Experiments 1–3. It clearly shows that the time lags in stimulus presentation are stable and constant even when the stimuli have a high rendering workload in VR environments. In Experiments 4A and B, the accuracy and precision were not affected by the complex visual stimuli. Generally, the gray-to-gray transitions on LCD have additional time lags because the raise time to peak luminance (i.e., 90% gray level) and fall time to the low luminance (i.e., 10% gray level) take more time to change compared with the black-to-white transitions (Boher et al., 2007). In the present study, the time lags between gray-to-gray and black-to-white transitions did not differ (i.e., 18 ms). The stimulus duration and its jitter (less than 1 ms) were also the same in black-to-white transitions. As OLED that has faster response time than LCD is used in modern VR HMDs, the time lags to present visual stimuli in gray-to-gray transitions may vary little, suggesting that the millisecond accuracy and precision are accomplished. This is supported by the results of Experiment 4B. While the VR scene in Experiment 4B was a complex visual stimulus with high rendering workload, where the luminance levels of each pixel on HMDs were not uniform as gray screens due to various RGB values of textures in the scene, the accuracy and precision did not vary (same as in black-to-white and gray-to-gray transitions). In 99.99-ms duration, the jitter of both HMDs (approximately 1 ms in HTC, 3 ms in Oculus) was slightly bigger than in the shorter durations (less than 1 ms). If the stimulus duration is longer (over 100 ms), the fall time from the various luminance level to the uniform black blank (lowest luminance level) on each pixel may vary slightly, depending on the VR HMDs, although the average duration is accurate.

The results of Experiment 5 also show that the accuracy and precision do not vary even when a complex auditory stimulus, which is more typical for VR experiments used in VR HMDs, is presented. The constant time lags of 38 or 58 ms with 3-ms jitter occur, depending on the VR HMDs. Consistent with the results of Experiment 1 (pure tone sound), the complex sound was also distorted in the short durations of 11.11 and 22.22 ms, indicating that at least 30-ms duration is required for accurate stimulus presentation in general VR experiments. Besides the time lags, in 99.99-ms duration, the jitters of HTC and Oculus HMDs were remarkably improved (under 4 ms), consistent with the results of Experiment 2, confirming that the 100-ms duration is sufficient for VR general experiments using realistic BGM or sound effects.

Importantly, as shown in Experiments 6A and B, the accuracy and precision of audio–visual stimulus presentation of complex stimuli are consistent with that of simple stimuli in Experiment 3. These results obviously confirm that the SOA between auditory and visual stimuli in VR experiment controlled by Python environments is constant but strongly depends on VR devices (19 ms in the HTC HMD and 39 ms in the Oculus HMD with 3-ms jitter). As described above, researchers can adjust these SOA by presenting the stimuli a few frames faster in the program. For instance, in VR experiments with HTC HMD, the auditory stimulus should be presented two frames (22 ms) faster than the visual stimulus, enabling only 3 ms SOA between auditory and visual stimuli. This small SOA is quite sufficient for general VR experiments as well as standard 2D experiments.

If there is no measurement and validation before the experiments, unsuitable SOA among visual, auditory, and trigger stimuli for external devices would be crucial for eye tracking and multisensory research in VR. Eye tracking technology is commonly used in VR research to examine how visual cognition works in 3D environments. For instance, when the experimental task is involved in the onset or offset of eye movements synchronized with stimuli in a trial sequence, the time lag between the actual onset of eye movements and triggered time (time stamp) in eye tracker devices must be confirmed, as a previous study on the temporal and spatial quality of HMD’s eye tracking suggests that the accuracy and precision also depend on the eye tracker device in HMDs (Lohr et al., 2019). In multisensory research on VR, multiple types of information such as auditory, visual, and haptic stimuli can be used simultaneously Burdea et al., 1996; Wilson & Soranzo, 2015). Especially in neuro-rehabilitation studies, haptic devices such as VR gloves are potential keys to providing kinesthetic or tactile stimulation for participants in VR (Demain et al., 2013). When haptic feedback synchronized with visual and auditory information is used for rehabilitation, the time lags among stimuli should be validated as much as possible. The time lag of the auditory stimulus from the visual stimulus can be used for adjusting the accuracy of haptic devices because haptic feedback is generated by frequency and amplitude in the same manner as an auditory stimulus if the haptic stimulus is controlled by vibration interfaces. However, when researchers perform experiments requiring strict millisecond control of the stimulus, the time lags must also be adjusted before the experiments to improve the unsuitable time/timing gap among multiple stimuli, testing their own VR devices.

## Limitations

The limitations of this study were the operating systems, the gray-to-gray transition levels, and the participants’ response times. We used the Vizard software for stimulus presentation in VR to systematically investigate the accuracy and precision of stimulus generation of modern VR HMDs in Python 2 and 3 environments. However, it only supports Windows operating systems. Previous studies on non-VR environments have indicated that accuracy and precision vary depending on which operating systems are used for software (Bridges et al., 2020; Krause & Lindemann, 2013; Tachibana & Niikuni, 2017). Moreover, the version of Apple operating system (current macOS vs. older OS X) seems to cause critical differences in stimulus presentation (Bridges et al., 2020). The stimulus presentation should be tested in VR experiments across all common operating systems with different versions by other VR software that has cross-platform support, although it is difficult to evaluate those directly as there are limited options for graphics card that can be used in Apple computers.

In Experiments 4A and 6A, we measured the accuracy and precision using only 10–90% gray-to-gray transitions, which are general gray levels for the test (Boher et al., 2007; Liang & Badano, 2006). A previous study has shown that other gray-level transitions such as 30–70% levels have a different temporal resolution for the presentation of visual stimuli on Gaming LCD (Poth et al., 2018). Due to the specific limitation of sensor threshold on BBTK, the luminance changes between 30% and 70% on VR HMDs could not be measured as precisely. BBTK, which we used as the evaluation tool in the present study, is a special device with 36 channels (6-kHz down-sampling rate) used to test the onset and offset timing accuracy and precision of stimulus synchronization with TTL triggers, enabling researchers to test various stimulus presentations simultaneously for a long period (1000 or more trial times is the common setting in human behavior studies). In contrast, a typical oscilloscope has 2–4 channels (100-MHz high sampling rate) and can be used only for a very short measurement period due to its low internal memory capacity (https://www.blackboxtoolkit.com/faq.html). Although BBTK is sufficient to measure the general timing accuracy and precision of stimulus presentation as in previous studies (Bridges et al., 2020), more specific measurements of gray-to-gray luminance changes on modern VR HMDs (i.e., OLED) are required by an oscilloscope to examine how specific gray-to-gray transitions affect the timing accuracy on OLED compared to LCD for the psychological and neuroscientific research.

The accuracy and precision of the response time should be tested using VR response devices. Participants in VR experiments may respond in several ways, such as keyboards, response pads, joysticks, VR controllers, and VR gloves. While the response pads and gaming keyboards commonly used in behavior experiments have high accuracy and precision when collecting participants’ data, the accuracy and precision of VR response devices are not well known. In particular, recent VR gloves such as Manus, which has haptic feedback, can be a key for dynamic response methods in VR studies. In future studies, a comparison between traditional and VR response devices should be conducted to reveal how reliable VR response devices are in terms of collecting participants’ real-time data.

## Conclusions

In summary, although there are time lags for each visual, auditory, and audio–visual stimulus presentation in modern VR HMDs, the accuracy and precision are stable across various stimulus types and capable of achieving millisecond time and timing control by a few frame adjustments. While the visual stimulus has a constant 18-ms time lag with less than 1-ms jitter, the auditory stimulus has a time lag of 37 ms on HTC Vive Pro and 58 ms on Oculus Rift with 4-ms jitter that depend on VR HMDs. These accuracies and precisions are robustly equal in both Python 2 and 3 environments for VR experiments, enabling the establishment of a more reliable experimental set up for psychological and neuroscientific research using various Python software. This study is also beneficial for researchers and developers who apply VR technologies for real-time communication where a number of people (and VR avatars) interact through VR (e.g., VR chat or meeting), as well as studies on rehabilitation tools that require high timing accuracy for recording biological data, improving unsuitable latency.

## Figures and Tables

**Table 9:**
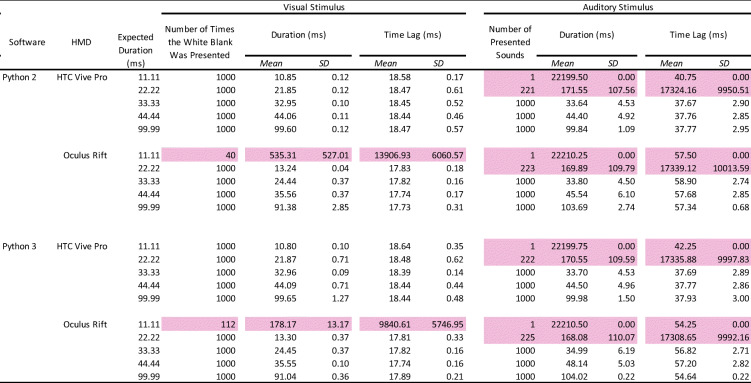
Summary of the presented number, mean duration, and onset lag with standard deviation in Experiment 3

The pink-highlighted data represent no accuracy of stimulus presentation. HMD: head-mounted display. SD: standard deviation.

**Table 10:**
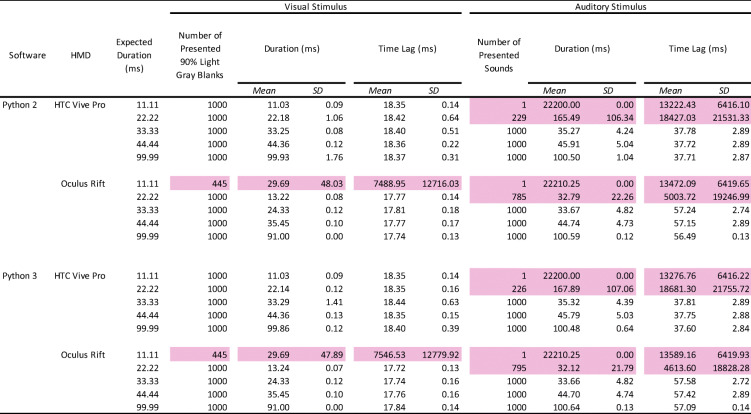
Summary of the presented number, mean duration, and onset lag with standard deviation in Experiment 6A

The pink-highlighted data represent no accuracy of stimulus presentation. HMD: head-mounted display. SD: standard deviation.

**Table 11:**
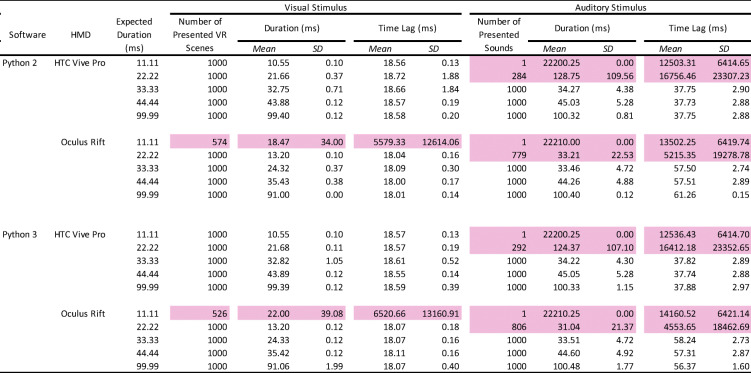
Summary of the presented number, mean duration, and onset lag with standard deviation in Experiment 6B

The pink-highlighted data represent no accuracy of stimulus presentation. HMD: head-mounted display. SD: standard deviation.

## Data Availability

The experiment code and raw data are available at Open Science Framework: https://osf.io/mp3n2/.

## References

[CR1] Boher, P., Glinel, D., Leroux, T., Bignon, T., & Curt, J. N. (2007). Relationship between LCD Response Time and MPRT. *In SID Symposium Digest of Technical Papers*, 38 (1), 1134–1137.

[CR2] Borrego A, Latorre J, Alcañiz M, Llorens R (2018). Comparison of Oculus Rift and HTC Vive: feasibility for virtual reality-based exploration, navigation, exergaming, and rehabilitation. Games for health journal.

[CR3] Bridges D, Pitiot A, MacAskill MR, Peirce JW (2020). The timing mega-study: comparing a range of experiment generators, both lab-based and online. PeerJ.

[CR4] Burdea G, Richard P, Coiffet P (1996). Multimodal virtual reality: Input-output devices, system integration, and human factors. International Journal of Human–Computer Interaction.

[CR5] Cipresso P, Giglioli IAC, Raya MA, Riva G (2018). The past, present, and future of virtual and augmented reality research: a network and cluster analysis of the literature. Frontiers in psychology.

[CR6] Choi SW, Lee S, Seo MW, Kang SJ (2018). Time sequential motion-to-photon latency measurement system for virtual reality head-mounted displays. Electronics.

[CR7] Cooper EA, Jiang H, Vildavski V, Farrell JE, Norcia AM (2013). Assessment of OLED displays for vision research. Journal of Vision.

[CR8] Dalmaijer ES, Mathôt S, Van der Stigchel S (2014). PyGaze: An open-source, cross-platform toolbox for minimal-effort programming of eyetracking experiments. Behavior Research Methods.

[CR9] Demain S, Metcalf CD, Merrett GV, Zheng D, Cunningham S (2013). A narrative review on haptic devices: relating the physiology and psychophysical properties of the hand to devices for rehabilitation in central nervous system disorders. Disability and Rehabilitation: Assistive Technology.

[CR10] Elze T (2010). Achieving precise display timing in visual neuroscience experiments. Journal of Neuroscience Methods.

[CR11] Foerster RM, Poth CH, Behler C, Botsch M, Schneider WX (2019). Neuropsychological assessment of visual selective attention and processing capacity with head-mounted displays. Neuropsychology.

[CR12] Garaizar P, Vadillo MA (2014). Accuracy and Precision of Visual Stimulus Timing in PsychoPy: No Timing Errors in Standard Usage. PLoS ONE.

[CR13] Ghirardelli, T. G., & Scharine, A. A. (2009). Auditory-visual interactions. *Helmet-mounted displays: Sensation, perception and cognition issues*, 599–618.

[CR14] Ghodrati M, Morris AP, Price NSC (2015). The (un)suitability of modern liquid crystal displays (LCDs) for vision research. Frontiers in Psychology.

[CR15] Kim KS, Wang H, Max L (2020). It's About Time: Minimizing Hardware and Software Latencies in Speech Research With Real-Time Auditory Feedback. Journal of Speech, Language, and Hearing Research.

[CR16] Krause F, Lindemann O (2013). Expyriment: A Python library for cognitive and neuroscientific experiments. Behavior Research Methods.

[CR17] Le Chénéchal, M., & Chatel-Goldman, J. (2018). HTC Vive Pro time performance benchmark for scientific research. *ICAT-EGVE 2018*, Limassol, Cyprus. ffhal-01934741f. https://hal.archives-ouvertes.fr/hal-01934741/document.

[CR18] Liang H, Badano A (2006). Precision of gray level response time measurements of medical liquid crystal display. Review of scientific instruments.

[CR19] Lohr, D. J., Friedman, L., & Komogortsev, O. V. (2019). Evaluating the data quality of eye tracking signals from a virtual reality system: case study using SMI’s eye-tracking HTC vive. arXiv preprint arXiv:1912.02083.

[CR20] Loomis JM, Blascovich JJ, Beall AC (1999). Immersive virtual environment technology as a basic research tool in psychology. Behavior Research Methods, Instruments, & Computers.

[CR21] Mathôt S, Schreij D, Theeuwes J (2012). OpenSesame: An open-source, graphical experiment builder for the social sciences. Behavior Research Methods.

[CR22] Muller E, Bednar JA, Diesmann M, Gewaltig M-O, Hines M, Davison AP (2015). Python in neuroscience. Frontiers in Neuroinformatics.

[CR23] Niehorster DC, Li L, Lappe M (2017). The Accuracy and Precision of Position and Orientation Tracking in the HTC Vive Virtual Reality System for Scientific Research. I-Perception.

[CR24] Pan X, de Hamilton AFC (2018). Why and how to use virtual reality to study human social interaction: The challenges of exploring a new research landscape. British Journal of Psychology.

[CR25] Parsons TD (2015). Virtual Reality for Enhanced Ecological Validity and Experimental Control in the Clinical, Affective and Social Neurosciences. Frontiers in Human Neuroscience.

[CR26] Peirce J, Gray JR, Simpson S, MacAskill M, Höchenberger R, Sogo H (2019). PsychoPy2: Experiments in behavior made easy. Behavior Research Methods.

[CR27] Plant RR, Hammond N, Turner G (2004). Self-validating presentation and response timing in cognitive paradigms: How and why?. Behavior Research Methods, Instruments, & Computers.

[CR28] Plant RR (2016). A reminder on millisecond timing accuracy and potential replication failure in computer-based psychology experiments: An open letter. Behavior Research Methods.

[CR29] Poth CH, Foerster RM, Behler C, Schwanecke U, Schneider WX, Botsch M (2018). Ultrahigh temporal resolution of visual presentation using gaming monitors and G-Sync. Behavior research methods.

[CR30] Reimers, S., & Stewart, N. (2016). Auditory presentation and synchronization in Adobe Flash and HTML5/JavaScript Web experiments. *Behavior Research Methods*, 1–12. 10.3758/s13428-016-0758-5 10.3758/s13428-016-0758-5 PMC5003904 27421976

[CR31] Rizzo AA, Schultheis M, Kerns KA, Mateer C (2004). Analysis of assets for virtual reality applications in neuropsychology. Neuropsychological Rehabilitation.

[CR32] Rhoads, S. (2019). A brief introduction to Python for psychological science research. *Psychological Science Agenda*. http://www.apa.org/science/about/psa/2019/07/python-research

[CR33] Tachibana R, Niikuni K (2017). Evaluation of stimuli timing accuracy with Expyriment under OS X. International Journal of Psychology and Neuroscience.

[CR34] Wiesing M, Fink GR, Weidner R (2020). Accuracy and precision of stimulus timing and reaction times with Unreal Engine and SteamVR. PLoS ONE.

[CR35] Wilson, C. J., & Soranzo, A. (2015). The Use of Virtual Reality in Psychology: A Case Study in Visual Perception. *Computational and Mathematical Methods in Medicine*, *vol. 2015*, 1–7. 10.1155/2015/151702 10.1155/2015/151702 PMC4538594 26339281

